# A Review of Oxygen Use During Chest Compressions in Newborns—A Meta-Analysis of Animal Data

**DOI:** 10.3389/fped.2018.00400

**Published:** 2018-12-18

**Authors:** Catalina Garcia-Hidalgo, Po-Yin Cheung, Anne Lee Solevåg, Maximo Vento, Megan O'Reilly, Ola Saugstad, Georg M. Schmölzer

**Affiliations:** ^1^Faculty of Science, University of Alberta, Edmonton, AB, Canada; ^2^Centre for the Studies of Asphyxia and Resuscitation, Neonatal Research Unit, Royal Alexandra Hospital, Edmonton, AB, Canada; ^3^Department of Pediatrics, University of Alberta, Edmonton, AB, Canada; ^4^Department of Pediatric and Adolescent Medicine, Akershus University Hospital, Lørenskog, Norway; ^5^Health Research Centre, University and Polytechnic Hospital La Fe, Valencia, Spain; ^6^Division of Neonatology, University and Polytechnic Hospital La Fe, Valencia, Spain; ^7^Spanish Maternal and Infant Health and Development Network, National Network, Spain; ^8^Department of Pediatric Research, University of Oslo, Oslo, Norway

**Keywords:** infants, newborn, neonatal resuscitation, chest compressions, oxygen, asphyxia

## Abstract

**Background:** International consensus statements for resuscitation of newborn infants recommend provision of 100% oxygen once chest compressions are required. However, 100% oxygen exacerbates reperfusion injury and reduces cerebral perfusion in newborn babies.

**Objective:** We aimed to establish whether resuscitation with air during chest compression is feasible and safe in newborn infants compared with 100% oxygen.

**Methods:** Systematic search of PubMed, Google Scholar and CINAHL for articles examining variable oxygen concentrations during chest compressions in term newborns.

**Results:** Overall, no human studies but eight animal studies (*n* = 323 animals) comparing various oxygen concentrations during chest compression were identified. The pooled analysis showed no difference in mortality rates for animals resuscitated with air vs. 100% oxygen (risk ratio 1.04 [0.35, 3.08], I^2^ = 0%, *p* = 0.94). ROSC was also similar between groups with a mean difference of −3.8 [−29.7–22] s, I^2^ = 0%, *p* = 0.77. No difference in oxygen damage or adverse events were identified between groups.

**Conclusions:** Air had similar time to ROSC and mortality as 100% oxygen during neonatal chest compression. A large randomized controlled clinical trial comparing air vs. 100% oxygen during neonatal chest compression is warranted.

## Introduction

Approximately, 3% of infants require respiratory assistance at birth and 0.1% require chest compressions (CC) (1, 2). During respiratory support of term and near term newly born infants air (21% oxygen) should be given as trials and meta-analyses reported a significant reduction in mortality in infants resuscitated with air (relative risk 0.71 [95% CI 0.54 to 0.94], risk difference −0.05 [−0.08 to −0.01]) (3, 4), which is also reflected in the neonatal resuscitation guidelines (1, 2). Oxygen (O_2_) use in the delivery room is associated with potential adverse effects; hyperoxia slows cerebral blood flow, brief periods of 100% O_2_ causes long-term reductions in cerebral blood flow. High concentrations of O_2_ lead to generation of oxygen free radicals, which have a role in reperfusion/reoxygenation injury after asphyxia especially to oxyregulator tissues such as myocardium (5–8). Thus, air might be a more appropriate gas than 100% O_2_.

If starting with air has been unsuccessful, the current resuscitation guidelines suggest to titrate oxygen and increase oxygen to 100% once chest compressions are started (1, 2). However, there is lack of supporting evidence of the beneficial effects of either titrating oxygen or using 100% O_2_ during cardiopulmonary resuscitation (CPR). The aim of oxygen use during resuscitation is to reactivate mitochondrial activity and energy provision and prevent tissue damage from oxygen deprivation during asphyxia while avoiding adverse effects of oxidative stress on the respiratory system and cerebral circulation; and tissue damage from oxygen free radicals (9).

If air is equally effective as 100% O_2_ in newborn infants requiring CC, the use of air instead of 100% O_2_ could reduce morbidity and mortality in asphyxiated infants. The aim of the meta-analysis was to compare the efficacy of air compared to 100% O_2_ during chest compression in the resuscitation in newborn infants immediately after birth. Further aims included assessment of oxidative stress and inflammatory markers using air vs. 100% O_2_.

## Methods

We searched PubMed, Google Scholar, and CINAHL using the following search terms (last searched on June 6, 2018, Appendix 1): “infant,” “newborn,” “resuscitation,” “chest compression,” “oxygen,” and “delivery room.” Publications were assessed based on title, abstract, and methods. Studies were included if they compared different oxygen concentrations during CC. Studies were excluded when no CC were performed, or if they did not define the infants as newborn. The initial search was aimed to only identify human trials. However, no human trials were identified and therefore the search was expanded to include animal studies. A manual search through the references of the obtained articles was additional performed.

### Study Selection

Two reviewers (CGH and GMS) independently reviewed citations for selection. Studies were included in the review if they met the following criteria: randomized controlled trial; comparing use air vs. 100% O_2_ during neonatal CPR; and presented the outcomes of either death or ROSC. Our primary outcome measure was mortality during neonatal CPR. Secondary outcomes included time to ROSC, oxygenation, and indicators of organ injury or damage. Full articles for potentially relevant studies were retrieved and independently assessed for their eligibility using a standardized data collection form. We also aimed to identify and if available include multiple publications describing the same study. Authors were contacted for data on return of spontaneous circulation (ROSC) as they were only reported as median (IQR) in the respective articles. No language restrictions were applied. Discrepancies regarding inclusion were resolved with another member of the review team (ALS).

### Data Extraction

Data were recorded using a standardized data collection form to record study design and methodological characteristics, patient characteristics, interventions, and outcomes thereof, including their RR (95% CI). Data extraction was independently performed by two investigators (GMS, CGH) and discrepancies were resolved in consultation with another member of the review team (ALS).

### Statistical Analysis

The principal summary measures were RR (95% CI) for dichotomous outcomes. Heterogeneity was explored using a chi-square test, and the quantity of heterogeneity was measured using the I^2^ statistic. We summarized RR estimates using random-effects models. Analyses were performed in RevMan version 5.3 (Cochrane Collaboration, 2014). All *p*-values are 2-tailed.

## Results

**Figure 1:**
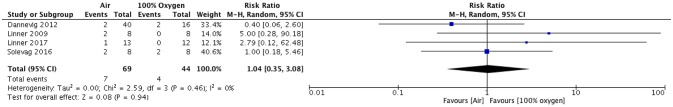
Mortality in piglets resuscitated with either Air or 100% oxygen during neonatal cardio-pulmonary resuscitation.

We did not identify any clinical human study on the subject. Therefore, all results come from controlled animal models. Six (75%) studies used a swine model (10–15), and two (25%) an ovine model (16, 17). Included studies assessed mortality (Figure 1), ROSC and/or circulatory recovery, oxygenation, severity of oxygen injury, and adverse events caused by oxygen. Table 1 presents all identified animal studies, intervention groups, and primary outcome, and conclusion (Table 1). Table 2 presents the pH, pCO_2_, base excess, and lactate prior the start of resuscitation (Table 2). Studies resuscitated animals with air or 100% O_2_ during CPR. All piglet studies used a post-transitional model (18), while the lamb studies used a fetal-to-neonatal transitional model (18). Dannevig et al. (10, 11) assessed brain and lung inflammation/injury in asphyxiated newborn piglets resuscitated with 21 vs. 100% O_2_, different compression to ventilation ratios (C:V) and variable durations of initial positive pressure ventilation. Alsaleem et al. (17) resuscitated asphyxiated newborn lambs with 21 or 100% O_2_ to evaluate cerebral O_2_ delivery and time to ROSC. The other ovine study, by Perez-de-Sa et al. (16) resuscitated asphyxiated lambs with air, 100% O_2_ for 3 min or 100% O_2_ for 30 min and assessed brain tissue oxygen tension. Using a newborn piglet model a study by Linner et al. (12) also tested resuscitation with air vs. 100% O_2_ for 3 or 30 min after asphyxia to compare time to ROC and cerebral oxygenation. A second study by Linner et al. (13) compared time to ROSC in asphyxiated newborn piglets with air or 100% O_2_ when ventilation is inadequate. Solevåg et al. compared time to ROSC in asphyxiated newborn piglets resuscitated with air and 100% O_2_ while an additional study compared 3:1 C:V vs. continuous CC with asynchronous ventilation (CCaV) in addition to air vs. 100% O_2_ and assessed time to ROSC and oxidative stress(14, 15).

**Table 1:** Oxygen in experimental animal models of neonatal cardiac arrest.

**Study**	**Experimental model**	**Groups**	**Outcomes**	**Conclusions**
**PORCINE**				
Dannevig et al. (10)	Porcine (14–34 h old) Asphyxial cardiac arrest Resuscitation: PPV, CC + ventilation (C:V), oxygen, epinephrine	PPV time pre-CC: 30s vs. 60s vs. 90s C:V ratios: 3:1 vs. 9:3 vs. 15:2 Oxygen: 21 vs. 100%	S100 in CSF: higher with 90s PPV than 30s or 60s IL-6 and TNF-α in CSF: higher with 30s PPV than 60s MMP-2 and ICAM-1 in CSF: higher with 30s PPV than 60s C:V ratios or oxygen percentage did not modulate inflammatory markers	Resuscitation should include a ventilation period longer than 30 s before commencing chest compressions
Dannevig et al. (11)	Porcine (12–36 h old) Asphyxial cardiac arrest Resuscitation: PPV, CC + ventilation (C:V), oxygen, epinephrine	PPV time pre-CC: 30s vs. 60s vs. 90s C:V ratios: 3:1 vs. 9:3 Oxygen: 21 vs. 100%	IL-8 and TNF-α in BAL and ICAM-1 and MMP-2 in lung tissue: higher with 30s PPV than 60s C:V ratios or oxygen percentage did not modulate inflammatory markers	Resuscitation with longer initial ventilation prior to the start of chest compressions should be considered
Linner et al. (12)	Porcine (12–36 h old) Asphyxial cardiac arrest Resuscitation: ventilation + oxygen, CCCM (if needed), epinephrine	Oxygen: air vs. 3-min 100% O_2_ vs. 30-min 100% O_2_	No significant differences in resuscitation times, arterial pressure responses, time until CrS_O2_ reached 30%, or time until Pbt_O2_ increased by 0.1 kPa from its nadir	Pure oxygen does not accelerate the recovery of circulation or of cerebral oxygenation, and resuscitation using air should be adequate provided the lungs are normal and easy to ventilate
Linner et al. (13)	Porcine (12–36 h old) Asphyxial cardiac arrest Resuscitation: ventilation + oxygen, CCCM (if needed), epinephrine	1-breath per min during first 10-min ventilation: air vs. 100% O_2_	Need for CCCM at 10-min: air 8/8 vs. 100% O_2_ 0/8 (*p* < 0.001) Median time to ROSC: air 845-s vs. 100% O_2_ 60-s (*p* < 0.001) No reported brain tissue hyperoxia	During inadequate ventilation, one oxygen breath reduced the time to ROSC
Solevåg et al. (14)	Porcine (1–3 days old) Asphyxial cardiac arrest Resuscitation: ventilation, CC + oxygen, epinephrine	Air + 3:1 C:V vs. 100% O_2_ + 3:1 C:V vs. Air + CCaV vs. 100% O_2_ + CCaV	No significant differences in time to ROSC or mortality Higher LV stroke volume post-ROSC and less myocardial oxidative stress in Air groups vs. 100% O_2_ groups Lower mean arterial BP post-ROSC and higher myocardial lactate in CCaV groups vs. 3:1 C:V groups	Resuscitation with air may reduce myocardial oxidative stress and improve cardiac function compared to 100% oxygen Resuscitation using CCaV may impair tissue perfusion compared to 3:1 C:V
Solevåg et al. (15)	Porcine (1–3 days old) Asphyxial cardiac arrest Resuscitation: ventilation, CC + oxygen, epinephrine	Air vs. 100% O_2_	ROSC: Air 16/16 vs. 100% O_2_ 14/16 Median time to ROSC (IQR): air 150-s (115–180) vs. 100% O_2_ 135-s (113–168); *p* = 0.80 No significant differences in temporal changes in mean arterial pressure, HR, pH, pCO_2_, IL-1β, or lactate/pyruvate ratios. Higher systemic and regional cerebral oxygen saturations in 100% O_2_ group	Resuscitation with room air seems to be as safe and effective as the use of 100% oxygen
**SHEEP**				
Perez-de-Sa et al. (16)	Lamb (near term, 140–141/145–150 days of gestation) Asphyxial cardiac arrest Resuscitation: ventilation + oxygen, CCCM + epinephrine (if needed)	Oxygen: air vs. 3-min 100% O_2_ vs. 30-min 100% O_2_	Pbt_O2_ median (range): air 2.9 (0.8–5.4) kPa vs. 3-min 100% O_2_ 4.2 (2.9–46) kPa vs. 30-min 100% O_2_ 56 (30–61) kPa HR and BP increased equally fast in all groups	Ventilation with air will restore circulation as fast as when pure oxygen is used, if ventilation is unobstructed and the lung are normal
Alsaleem et al. (17)	Fetal lamb Asphyxial cardiac arrest Resuscitation: ventilation, CC + oxygen, epinephrine	Oxygen during CC: air vs. 100% O_2_	ROSC: air 7/7 vs. 100% O_2_ 6/6 No significant differences in time to ROSC, number of epinephrine doses, carotid artery blood flow, SpO_2_, PaO_2_, CaO_2_ or O_2_ delivery to the brain during CC Higher PaO_2_ immediately post-ROSC in 100% O_2_ group (*p* = 0.046)	Resuscitation with 100% oxygen does not enhance oxygen delivery to the brain or time to ROSC but increases PaO_2_ levels post-ROSC Weaning and titrating FiO_2_ immediately after ROSC to maintain preductal saturations in the 85–95% range is recommended

*BAL, bronchoalveolar lavage fluid; BP, blood pressure; CaO_2_, arterial oxygen content; CC, chest compressions; CCaV, continuous chest compressions with asynchronous ventilation; CCCM, closed chest cardiac massage; CrS_O2_, regional oxygen saturation; CSF, cerebral spinal fluid; C:V, compression to ventilation ratio; FiO_2_, fraction of inspired oxygen; HR, heart rate; O_2_, oxygen; PaO_2_, partial pressure of oxygen; Pbt_O2_, brain tissue oxygen tension; pCO_2_, partial pressure of carbon dioxide; P_O2_, oxygen tension; PPV, positive pressure ventilation; SpO_2_, blood oxygen saturation*.

**Table 2:** Indicators of sickness before resuscitation with variable oxygen concentration during ventilation paired with CC during infant resuscitation.

	**pH**		**pCO**_****2****_ **(kPa)**		**Base excess (mmol/L)**		**Lactate (mmol/L)**	
	**Air (21%)**	**100% O_**2**_**	**Air (21%)**	**100% O_**2**_**	**Air (21%)**	**100% O_**2**_**	**Air (21%)**	**100% O_**2**_**
Dannevig et al. (11)	6.7		4.5–6		Below−30		N/A	
Linner et al. (13)	6.8 (6.7–6.9)	6.7 (6.5–6.8)	15 (10–20)	18 (13–19)	−17 (−21 – to 7)	−16 (−22 to – 11)	15 (10–19)	16 (11–20)
Linner et al. (12)	6.7 (6.6–6.7)	6.70 (6.6–6.8)	21.4 (20.2–23.8)	19.6 (17.3–21.6)	−18.9 (−21.5 to −18.3)	−20.4 (−22.0 to −18.4)	N/A	
Solevåg and Nakstad (15)	6.6 (0.2)	6.7 (0.2)	10.3 (2.4)	9.3 (2.1)	−28.6 (5.6)	−28.0 (6.1)	9.6 (4.8)	7.5 (4.2)
Solevåg et al. (14)	6.6 (6.5–6.7)	6.5 (6.5–6.7)	12.8 (11.6–14.5)*	10.9 (7.9–13.3)*	N/A		18.3 (14.1–18.9)	17.6 (15.0–19.9)
Perez-de-Sa et al. (16)	N/A		17 (16–19)	17 (14–19)	−14 (−21 to −8)	−12 (−15 to −8)	N/A	

*Reported as median (IQR) or mean (SD)*,

* *presented as mmHg in the original article, converted to kPa, Dannevig et al. (10) and Alsaleem et al. (17) not reported as data was not available*.

### Mortality

For the pooled analysis, the study by Solevåg et al. (15) was excluded because the study by Dannevig et al. (10) included the same piglets reported by Solevåg et al. (15). A total of 323 animals were included with an overall mortality of 17(5%). All animals in the ovine studies survived and therefore no pooled analysis was possible. For the outcome of mortality, the pooled analysis of the porcine studies showed no difference in mortality for piglets resuscitated with air vs. 100% oxygen (odds ratio 0.84 [0.26, 2.72], *p* = 0.77, I^2^ = 0%; Figure 1).

### Return of Spontaneous Circulation and Circulatory Recovery

**Figure 2:**
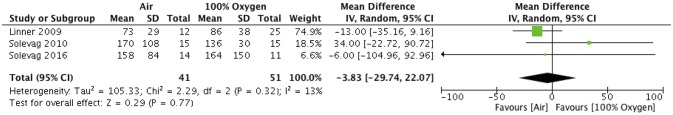
Time to return of Spontaneous Circulation in piglets resuscitated with either Air or 100% oxygen during neonatal cardio-pulmonary resuscitation.

Overall, the individual studies and the pooled analysis showed no difference in the time to ROSC with air vs. 100% O_2_ with a mean difference of −3.8 [−29.7–22] s, I^2^ = 0%, *p* = 0.77 (Figure 2). Solevåg et al. (15) randomly assigned piglets to receive air or 100% O_2_ during CPR after asphyxia induced asystole and reported similar median [interquartile range (IQR)] time to ROSC of 135 (113–168) s vs. 150 (115–180) s in the air and 100% group, respectively. A second study by Solevåg et al. (14) randomized newborn piglets to air vs. 100% O_2_ and 3:1 C:V vs. CCaV. Overall, no difference in time to ROSC between the groups were observed. Circulatory recovery including left ventricular stroke volume with air and 100% O_2_ was comparable 1.4 vs. 1.0 mL/kg and 0.8 vs. 0.5 mL/kg 30 min and 4 h after ROSC, respectively. Studies by Linner et al in 2009 and 2017 (12, 13) randomized piglets to air for the duration of resuscitation, 100% O_2_ for 3 min followed by air, or 100% O_2_ for 30 min followed by air after apnea induced cardiac arrest. Time until ROSC (heart rate >150 bpm) were similar among groups with median (IQR) times of 67 (60–76) s, 88 (76–126) s, and 68 (56–81) s in the air, 100% for three min group, or 100% for 30 min group, respectively.

Alsaleem et al. (17) induced cardiac arrest in fetal lambs by umbilical cord occlusion. The lambs were delivered and resuscitated with air then randomized to continue to receive air at the onset of CC or the O_2_ to be increased to 100%. There was no difference in mean (±SD) time to ROSC 211 (±145) s and 306 (±270) s in the air and 100% O_2_ group, respectively. Perez-de-Sa et al. (16), induced cardiac arrest in fetal lambs and randomized them to either air, 100% for the first 3 min, or 100% O_2_ during the first 30 min. If there was no sign of ROSC CC were initiated. No difference in time to a heart rate of 150 bpm was reported in all groups [68 (6–150) s, 107 (5–182) s and 58 (23–368) s]. These studies demonstrate that air is as effective as 100% O_2_ to achieve ROSC and circulatory recovery. However, recently Linner et al. demonstrated that when PPV is ineffective ROSC can be faster achieved using 100% O_2_ compared to air with 60 (11–120) s vs. 845 (611–death) s (*p* < 0.001) (13).

### Oxygenation

All studies confirmed the significant arterial hyperoxemia in the group of animals resuscitated with 100% oxygen. Using cerebral near-infrared spectroscopy, studies measured speed of recovery of brain oxygenation by how fast cerebral regional oxygen saturation reached 30% and when brain tissue oxygenation (PbtO_2)_ had increased 0.1 kPa from its nadir. Studies in near-term lambs reported higher PbtO_2_ with 100% O_2_, and significantly increased partial pressure of arterial oxygen levels immediately after ROSC compared to lambs who remained in air (165 ± 145 vs. 41 ± 16 mmHg), (*p* = 0.046) (17). However, throughout CC, no significant difference between blood oxygen saturation, partial pressure of arterial oxygen, arterial oxygen content, or O_2_ delivery to the brain between groups was observed (17). Perez-de-Sa et al. reported brain tissue oxygenation as measured by the partial pressure of oxygen in extracellular fluid (PbtO_2_) reaching a maximum of 56 kPa (420 mmHg) in the group initially ventilated with 100% O_2_ for 30 min, a level only previously seen in hyperbaric conditions(16). The groups receiving 100% O_2_ for 3 min had significantly lower PbtO_2_ peaking at 4.2 kPa (31.5 mmHg) while those receiving air peaked at 2.9 kPa (19.5 mmHg) (*p* = 0.002). The 2009 study by Linner et al. found no difference in time to recovery of brain oxygenation but observed arterial hyperoxemia 34 (30–41) kPa [255 (225–308) mmHg] by 2.5 min and peak PbtO_2_ values during resuscitation were higher in groups ventilated with 100% O_2_ [4.2 (3.3–5.4) kPa (31.5 (24.8–40.5) mmHg], 12 (6.4–15) kPa [90 (48–112) mmHg], and 25 (15–36) kPa [187.5 (112.5–270) mmHg], respectively) (12).

### Indicators of Organ Injury or Damage

There were two studies that also examined the tissue oxidative stress or damage when 100% O_2_ vs. air was used in the resuscitation after asphyxia induced cardiac arrest in newborn piglets. Dannevig et al. (10, 11) found no difference in brain or lung inflammatory markers (e.g., lactate, lactate/pyruvate, or IL-1) in the groups with 100% O_2_ or air. Dannevig et al. also examined damage associated with different C:V ratios and duration of initial PPV (10, 11), however these results were excluded as they were not within the scope of this review.

Damage caused by oxidative stress was measured in the piglet study assessing 100% O_2_ and air with 3:1 C:V or CCaV (14). The oxidized glutathione to glutathione ratio, a marker for oxidative stress, was significantly higher with 100% O_2_ than air, 0.14 (0.11–0.22) and 0.10 (0.08–0.11) (*p* = 0.005), respectively.

## Discussion

Current neonatal resuscitation guidelines recommend 100% O_2_ during CPR, however the most effective oxygen concentration in newborn infants remains controversial (2, 19, 20). Oxygen has been used in neonatal resuscitation for over 200 years (21). Its use spread rapidly in response to reports of brain damage in infants who had survived birth asphyxia (22). The inclusion of skin color in the Apgar score further contributed to an increased use of oxygen in the delivery room. The use of 100% oxygen was accepted based on experts' opinion despite a lack of experimental evidence. However, over the last decades the use of 100% O_2_ has been questioned as even a brief exposure to 100% O_2_ may be detrimental and several studies reported that air is as effective as 100% O_2_ (4, 23–27); Indeed, 21% O_2_ resulted in a significant reduction in mortality (28/284 vs. 60/321 [relative risk (RR) 0.71 (95% Confidence Interval (CI) 0.54 to 0.94), risk difference −0.05 (−0.08 to −0.01)] (3, 4), decreased time to first breath >3 min (102/321 vs. 71/288 [RR (95%CI) 0.78 (0.6–1.0)], and less Apgar scores < 7 at 5 min (107/659 vs. 70/616 [RR (95%CI) 0.71 (0.54–0.94)] when compared to 100% O_2_ (4, 4). This has led to a change in the 2010 neonatal resuscitation guidelines to start respiratory support with air and oxygen delivery titrated according to target oxygen saturations in term and near-term newborn infants (28). While these studies have examined oxygen use during respiratory support of term and near-term newborn infants no human study has compared air vs. 100% O_2_ during neonatal chest compression.

Our meta-analysis identified several animal studies comparing air vs. 100% O_2_ during chest compression in newborn animal models. Overall, air was as effective as 100% O_2_ during neonatal CPR to achieve ROSC [mean difference of −3.8 (−29.7–22) s, I^2^ = 0%, *p* = 0.77 (Figure 2)] and also had similar mortality rates between groups [(odds ratio 0.84 [0.26, 2.72], *p* = 0.77, I^2^ = 0%; Figure 1)].

High concentrations of O_2_ delivery during CPR generates O_2_-free radicals, which play major role in reperfusion/reoxygenation injury after asphyxia, especially to oxyregulatory tissues (e.g., myocardium) (28). During CPR 100% O_2_ causes significant arterial hyperoxemia and increased partial pressure of arterial oxygen levels immediately after ROSC (165 ± 145 vs. 41 ± 16 mmHg, *p* = 0.046) compared to resuscitation with air (17). However, this did not result in higher oxygenation at the brain. Furthermore, tissue oxygenation, and tissue/organ damage were not significantly different between air and 100% O_2_. Indeed, only the study by Solevåg et al. reported damage caused by oxidative stress in piglets resuscitated with 100% O_2_ compared to air. In the study by Solevåg et al. the oxidized glutathione to glutathione ratio, a marker for oxidative stress, was significantly higher with 100% O_2_ than air, 0.14 (0.11–0.22) and 0.10 (0.08–0.11) (*p* = 0.005), respectively (14). However, other studies did not report any difference in brain or lung inflammatory markers (e.g., lactate, lactate/pyruvate, or IL-1) in the groups with 100% O_2_ or air (10, 11). Based on these findings pure oxygen is not associated with damage to the nervous system or lungs any more than air but is a cause of oxidative stress. In addition, 100% O_2_ exposure at birth has been associated with increased risk of neonatal mortality and childhood cancer (29, 30).

## Limitations

Limitations of the current review among others include (i) only data from animal studies were included, (ii) the data should not be directly extrapolated to clinical practice, (iii) different animal models (e.g., piglets and sheep), (iv) transitional model (lambs delivered via cesarean section) vs. post-delivery model (piglets 1–3 days old), (v) induction of cardiac arrest by asphyxia or potassium chloride, or (vi) all animals were intubated with a tightly sealed endotracheal tube (except the latest by Linner et al.) to prevent any endotracheal tube leak (10–18, 31). These variations might have influenced the results; however, subgroup analysis would have been impossible given the small sample size of each study. In addition, results in preterm infants might differ due to their immature antioxidant defense system and increased likelihood to need resuscitation (32–34). Furthermore, not all studies were randomized and only the study by Solevåg et al. (14) adhered to the ARRIVE guidelines (35), which would have been a strength and could reduce potential bias.

## Future directions

Our results indicate that air during CC is safe and human trials are urgently needed. Human studies should compare air vs. 100% and examine effects of both oxygen concentrations to reduce exposure to hypoxia and hyperoxia (36). Alternatively, attempting to mimic the gradual rise in oxygen saturation of healthy term babies in the first 10 min after birth by titrating the concentration to the baby's saturation or using any intermediate options should be assessed in regards of benefits and harms (1, 2). Furthermore, any human trial should include long-term neurodevelopmental follow-up (29, 30).

## Conclusion

No human studies were identified and the results obtained are from animal models. The data suggest that using air instead of 100% oxygen during neonatal chest compression had comparable outcomes including time to return of spontaneous circulating and mortality. Hyperoxia and oxidative stress were significantly higher with 100% O_2_. Human trials comparing air vs. 100% and/or oxygen titration as an alternative to air or 100% oxygen during neonatal chest compression are urgently needed.

## Author Contributions

GS, P-YC, MO, and AS Conception and design; GS, CG-H, MO, MV, P-YC, AS, and OS Collection and assembly of data, analysis and interpretation of the data; GS, CG-H, MO, P-YC, AS, MV, and OS Drafting of the article; GS, CG-H, MO, P-YC, MV, AS, and OS; Critical revision of the article for important intellectual content, final approval of the article.

### Conflict of Interest Statement

The authors declare that the research was conducted in the absence of any commercial or financial relationships that could be construed as a potential conflict of interest.

## Abbreviations

3:1 C:V: Compression to Ventilation ratio
CPR: Cardiopulmonary resuscitation
CC: Chest compression
O_2_: Oxygen
PPV: Positive pressure ventilation
ROSC: Return of spontaneous circulation
IQR: Interquartile range
PbtO_2_: Brain tissue oxygenation
CCaV: Continuous CC with asynchronous ventilation.
