# Supplementary material for: A Review of Oxygen Use During Chest Compressions in Newborns—A Meta-Analysis of Animal Data
Source: Front Pediatr. 2018 Dec 18;6:400. doi: 10.3389/fped.2018.00400 (PMC6305367; doi:10.3389/fped.2018.00400)
Supplement: Supplementary file 1 [file Data_Sheet_1.pdf]

Appendix

PubMed

#1 Infant (n=1,228,218)

#2 Infants (n=1,158,752)

#3 Newborn (n=704,125)

#4 Resuscitation (n=113,064)

#5 Chest Compressions (n=2,121)

#6 Oxygen (n=577,174)

#7 Delivery Room (n=9,577)

#8 Neonatal (n=681,967)

#9 #1 AND #4 AND #5 AND #6 (#2 OR #3 OR #7 OR #8) [n=60, n=51, n=21, n=52)

Google Scholar

#1 Infant (n~3,130,000)

#2 Infants (n~2,080,000)

#3 Newborn (n~1,930,000)

#4 Resuscitation (n~1,110,000)

#5 Chest Compressions (n~69,600)

#6 Oxygen (n~4,800,000)

#7 Delivery Room (n~3,700,000)

#8 Neonatal (n~2,530,000)

#9 #1 AND #4 AND #5 AND #6 (#2 OR #3 OR #7 OR #8) [n~20,600, n~16,000, n~15,000, n~15,800)

CINAHL

#1 Infant (n=234,333)

#2 Infants (n=234,333)

#3 Newborn (n=111,026)

#4 Resuscitation (n=27,573)

#5 Chest Compressions (n=1,988)

#6 Oxygen (n=52,263)

#7 Delivery Room (n=2,486)

#8 Neonatal (n=49,290)

#9 #1 AND #4 AND #5 AND #6 (#2 OR #3 OR #7 OR #8) [n=17, n=13, n=7, n=11)
